# Overexpression of *PvCO1*, a bamboo CONSTANS-LIKE gene, delays flowering by reducing expression of the *FT* gene in transgenic *Arabidopsis*

**DOI:** 10.1186/s12870-018-1469-0

**Published:** 2018-10-12

**Authors:** Guohui Xiao, Bingjuan Li, Hongjun Chen, Wei Chen, Zhengyi Wang, Bizeng Mao, Renyi Gui, Xiaoqin Guo

**Affiliations:** 1 The State Key Laboratory of Subtropical Silviculture, Zhejiang A&F University, Hangzhou, 311300 China; 2 Institute of Biotechnology, College of Agriculture and Biotechnology, Zhejiang University, Hangzhou, 310029 China; 3 Zhejiang Provincial Collaborative Innovation Center for Bamboo Resources and High-efficiency Utilization, Zhejiang A&F University, Hangzhou, 311300 China

**Keywords:** *CONSTANS* (*CO*), Flowering time, Functional divergence, Flowering regulation, Bamboo, *Phyllostachys violascens*

## Abstract

**Background:**

In *Arabidopsis*, a long day flowering plant, *CONSTANS* (*CO*) acts as a transcriptional activator of flowering under long day (LD) condition. In rice, a short day flowering plant, *Hd1*, the ortholog of *CO*, plays dual functions in respond to day-length, activates flowering in short days and represses flowering in long days. In addition, alleles of *Hd1* account for ~ 44% of the variation in flowering time observed in cultivated rice and sorghum. How does it work in bamboo? The function of *CO* in bamboo is similar to that in *Arabidopsis*?

**Results:**

Two *CO* homologous genes, *PvCO1* and *PvCO2*, in *Phyllostachys violascens* were identified. Alignment analysis showed that the two PvCOLs had the highest sequence similarity to rice Hd1. Both *PvCO1* and *PvCO2* expressed in specific tissues, mainly in leaf. The *PvCO1* gene had low expression before flowering, high expression during the flowering stage, and then declined to low expression again after flowering. In contrast, expression of *PvCO2* was low during the flowering stage, but rapidly increased to a high level after flowering. The mRNA levels of both *PvCOs* exhibited a diurnal rhythm. Both PvCO1 and PvCO2 proteins were localized in nucleus of cells. PvCO1 could interact with PvGF14c protein which belonged to 14–3-3 gene family through B-box domain. Overexpression of *PvCO1* in *Arabidopsis* significantly caused late flowering by reducing the expression of *AtFT*, whereas, transgenic plants overexpressing *PvCO2* showed a similar flowering time with WT under LD conditions. Taken together, these results suggested that PvCO1 was involved in the flowering regulation, and PvCO2 may either not have a role in regulating flowering or act redundantly with other flowering regulators in *Arabidopsis*. Our data also indicated regulatory divergence between PvCOLs in *Ph. violascens* and CO in *Arabidopsis* as well as Hd1 in *Oryza sativa.* Our results will provide useful information for elucidating the regulatory mechanism of *COLs* involved in the flowering.

**Conclusions:**

Unlike to the *CO* gene in *Arabidopsis*, *PvCO1* was a negative regulator of flowering in transgenic *Arabidopsis* under LD condition. It was likely that long period of vegetative growth of this bamboo species was related with the regulation of PvCO1.

**Electronic supplementary material:**

The online version of this article (10.1186/s12870-018-1469-0) contains supplementary material, which is available to authorized users.

## Background

The transition from a vegetative phase to a reproductive phase is an important developmental switch in plants. This transition is controlled by several environmental and endogenous conditions [1, 2]. Different plant species have various mechanisms to regulate this process [3] and many, such as grasses, have distinct flowering habits.

Bamboo is one of the most important non-timber forests and belongs to the Poaceae. Unlike other plants in this family, such as rice, maize, and wheat, the flowering time of bamboo appears to be random. Some species have prolonged vegetative growth lasting decades before flowering and death. One such species is *Phyllostachys heterocycla*, a woody bamboo that has ecological, economic and cultural value [4]. Another economically important species, *Ph. violascens,* belongs to the same genus and has very similar genetic background with *Ph. heterocycla*. In this species, those elder plants at the age of 6 years would usually be harvested for gain yield of bamboo shoots. However, compared with *Ph. heterocycla*, the flowering pattern of *Ph. violascens* is variable. Its different individuals can flower at different times during the year. Some plants flower twice and more, some only once, some never flower even when they were harvested [5, 6]. Some young plants without leaves grow poorly but still flower and then die. There are some individual plants flower every year. Flowering duration can be 60 to 90 d. Many researchers have attempted to explain the factors controlling flowering. These factors include nutrition, climate, stress, and molecular mechanisms [7, 8]. Currently, studies on the molecular mechanism of bamboo flowering have focused on transcriptome sequencing and expression of genes involved in the developmental stages of flowering [4, 5, 9–14], while reports on the genes involved in floral induction are rare [6, 15–18]. Peng et al., [4] reported that repeat insertions in the regulatory region of most homologs encoding *CONSTANS* (*CO*), might result in low gene expression in *Ph. heterocycla*. And the *CONSTANS* (*CO*) gene was originally isolated as a photoperiodic floral promoter.

The *CO* gene in *Arabidopsis* plays a critical role in control of flowering time by directly activating the expression of target genes including *FLOWERING LOCUS T* (*FT*) which encodes a florigenic protein [19]. Overexpression of the *CO* gene accelerates *Arabidopsis* flowering regardless of photoperiod [20]. *CO* gene mutation results in delayed flowering under long-day (LD) conditions, but has no effect on flowering time under short-day (SD) [21]. *CO* encodes a B-box-type zinc-finger transcriptional factor with two B-box domains near the N-terminus and a CCT (*CO*, *CO-like*, and *TOC1*) domain near the C-terminus [21–23]. The B-box domain of CO is likely involved in protein-protein interactions and the CCT domain binds DNA [24–26]. The CO protein can bind to specific *cis*-elements in the *FT* promoter either by itself [24] or in a complex with CCAAT-binding factors [19, 26, 27] to regulate *FT* transcription. CO protein can also interact with specific 14–3-3 isoforms, 14–3-3 μ and ν proteins which belong to the family representing nodes of signal integration and cross talk, affecting photoperiodic flowering [28]. In rice, a SD plant, the *Hd1* gene, orthologous to *CO*, promotes flowering under SD conditions, but delays flowering in LD conditions [29, 30]. In addition, the mutant of *Se1*, allelic to *Hd1*, controlling photoperiod sensitivity, is also slightly later than its progenitor variety in heading date [29]. The wheat *TaHd-1* gene, also homologous to *CO*, can complement the function of rice *Hd1*: it also promotes heading under SD conditions, but delays it under LD conditions [31]. Overexpression of *LpCO* (from *Lolium perenne*) leads to early flowering in *Arabidopsis* [32]*.* The *PnCO* gene from *Pharbitis nil* can complement the *co* mutant of *Arabidopsis* [33]*.* Overexpression of *GmCOL1a*, *GmCOL1b*, *GmCOL2a* and *GmCOL2b* from soybean rescued the late flowering phenotype of *Arabidopsis co* mutant [34]. The alleles of *Hd1* account for ~ 44% of the variation in flowering time in cultivated rice and sorghum, suggesting *Hd1* plays an important role in flowering. Differences of *CO* gene expression are responsible for differences in flowering times [23]. CO is important to many plant species including poplar (*Populus* spp) [35], but its function remains unknown in non-model systems such as woody perennial bamboo species.

Whether the *COL* genes in bamboo have the influence on the flowering time are unclear and it is unknown if *COL* gene functions in bamboo are similar to those in *Arabidopsis*. In the present study, two homologous *CO* genes, *PvCO1* and *PvCO2*, were identified from *Ph. violascens*. Their expression patterns were analyzed and primary functions were characterized. The results give new insights into the understanding of the *COLs* genes involved in floral transition.

## Methods

### Plant materials

*Phyllostachys violascens* (Carriere) Riviere in this study were grown in the field under natural conditions on the campus of Zhejiang A&F University (30°14′N, 119°42′E). The mean annual temperature is 15.6 °C, with maximum and minimum temperatures of 41.7 °C and − 13.3 °C, respectively. The average length of sunshine in Lin’an is approximately 1,847 h per year. We chose those plants which flowered last year for sampling. Some of these plants flowered again from mid-March to mid-May and flowering lasts for 60 to 90 d.

To study expression of *PvCOLs* before, during, and after flowering, firstly, we sampled fully expanded, mature leaves from ten flowered plants at 5:00 pm on March 2, 2012. Ten days later, on March 12, we sampled leaves again from these ten flowered plants also at 5:00 pm. And then, we found 4 individual plants displayed flower bud and flowered again between on March 12 and 22, among which 3 were targeted for sampling. Thereafter, we collected leaves from these 3 flowering plants every 10 d until to May 31 at 5:00 pm because these three plants died between on May 31 and June 3. Day length increased from 11.5 h light on March 2 to 14 h light on May 31. Meanwhile, we immediately determined expression of target genes after collecting the leaf samples. Once the target gene was expressed, we also collected fully expanded, mature leaves from the same three flowering plants 8 times at 3 h intervals on March 30 (LD 12.5:11.5 h) and determined expression of target genes for diurnal expression analysis. The maximum and minimum temperatures were 15.7 °C and 13.3 °C on this day, respectively. We also sampled mature leaves, immature leaves, roots, stems and flower buds for determining the tissue-specific expression from 5 pm to 6 pm on April 13 (LD 13:11 h). The maximum and minimum temperatures on April 13 were 17.3 °C and 13.9 °C, respectively. All plant samples were stored at − 80 °C prior to further experiments.

Wild type (WT) and transgenic plants of *Arabidopsis thaliana* ecotype Columbia-0 (Col-0) were cultured in a room under ≈22 °C with LD (16 h light: 8 h dark) conditions. The light intensities is 200 umol/m .s.

### DNA and RNA procedures

Total genomic DNA was extracted from young leaves of *Ph. violascens* by the CTAB method and total RNAs were extracted from the collected samples using Trizol reagent (Invitrogen, US). To remove any residual genomic DNA from the preparation, the RNA was treated with RNase-free DNase I according to manufacturer instructions (Qiagen, Valencia, CA, US). The first-strand complementary DNA (cDNA) was synthesized using the Super Script III kit (Invitrogen, US), according to the manufacturer manual.

A pair of degenerate primers (TOHLF1/TOHLR2) was designed according to the conserved sequence of *CO* homologous genes from rice, maize and wheat, and used to amplify the partial DNA and cDNA of *PvCOL*. Based on the partial DNA sequence of *PvCOL*, the primers 5SP1, 5SP2, 5SP3, 3SP1, 3SP2 and 3SP3 used for genome walking amplification and the primers GSP1, GSP2, GSP3 and GSP4 used for rapid amplification of cDNA end (RACE) were designed in order to obtain the 5’ and 3’ terminal sequences of the *PvCOL* genes. The *PvCOL* DNA sequence containing the encoding region was assembled by a combination of the conserved sequence and the 5’ and 3’ terminal sequences. To obtain the full-length cDNA and genomic DNA sequence of *PvCOL*, two pairs of specific primers, PvCO1F and PvCO1R for *PvCO1*, PvCO2F and PvCO2R for *PvCO2*, were designed based on the assembled sequence and used for amplification. Detailed information on all primers used is listed in Additional file 1: Table S1. All of the amplified fragments were gel purified, ligated into the pMD18-T vector, transformed into the DH5a competent cells, and sequenced.

Real-time PCRs were performed according to the procedures of Guo et al., [6] and semi-quantitative PCR according to Putterill et al., [21]. Annealing temperature and the cycles of PCR were adjusted according to the primers and target genes. Primers used (PvCO1qexpF and PvCO1qexpR for *PvCO1*, PvCO2qexpF and PvCO2qexpR for *PvCO2*, PvActinqexpF and PvActinqexpR for *PvActin*, AtFTF and AtFTR for *AtFT*, AtActinF and AtActinR for *AtActin*) in real-time PCR experiments and primers used (PvCO1expF and PvCO1expR for *PvCO1*, PvCO2expF and PvCO2expR for *PvCO2*, ActinF and ActinR for *PvActin*) in semi-quantitative PCR. PCR primers are listed in Additional file 1: Table S1. When PCR analyses were conducted using plasmid DNA harboring complete *PvCO1* and *PvCO2* cDNA as templates, no cross-amplification was detected. In this study, *PvActin* and *AtActin* genes were used as reference genes for normalization because they have stable expression pattern [36].

### Bioinformatic analysis

The open reading frame (ORF) of *PvCOL* cDNA was determined using the ORF Finder (https://www.ncbi.nlm.nih.gov/orffinder/) and translated into the corresponding amino acid sequence. The conserved domain was predicted using CD search (http://www.ncbi.nlm.nih.gov/Structure/cdd/wrpsb.cgi). The predicted protein sequence alignments were performed via Clustalw, and the results of multiple sequence alignments were displayed by GENEDOC (http://iubio.bio.indiana.edu/soft/molbio/ibmpc/genedoc-readme.html). Phylogenetic analysis and statistical neighbor-joining bootstrap tests of the phylogenies were performed by MEGA version 5.0 (http://www.megasoftware.net/). Bootstraps with 1000 replicates for Poisson correction model were performed to assess node support. The information on 17 *COL* gene family members in *Arabidopsis* and 17 in rice was from Griffiths et al. [37] and Cockram et al. [38], respectively. The accession numbers for all these genes were listed in Additional file 2: Table S2.

To identify the *COL* genes in moso bamboo (*Ph. heterocycla*), we downloaded the genomic DNA sequence, predicted genes and protein sequences from Peng et al., [4] (http://202.127.18.221/bamboo/down.php), constructed local blast database using BioEdit software, and then used rice COL protein sequences as queries to perform BLASTp search with the expectation (e)-value threshold of 1.0e^− 30^. The candidate proteins containing B-box domains and CCT domains were predicted via the NCBI-CDD (http://www.ncbi.nlm.nih.gov/Structure/cdd/wrpsb.cgi). To ensure that these candidate proteins actually belong to the *COL* gene family, we deleted the proteins lacking the B-box domain or the CCT domain.

### Subcellular localization

The amplicons of the *PvCO1* and *PvCO2* CDS regions were inserted at the 5^’^end of a GFP gene driven by the CaMV35S promoter. The region corresponding to the PvCO1 C-terminal (Met269-Phe384) containing the CCT domain was amplified from the plasmid harboring complete *PvCO1* cDNA via PCR and fused to the 5^’^end of GFP. This fusion was called *PvCO1* (Cterm). Transient expression of the GFP fusions in onion epidermal cells were performed as previously described [39]. Then the onion epidermal cells were observed with a confocal laser scanning microscope.

### Transformation of *Arabidopsis*

The ORF of *PvCO1* and *PvCO2* amplified from the plasmids harboring complete *PvCO1* cDNA and complete *PvCO2* cDNA were purified and inserted into the pCAMBIA 1301 vectors, respectively, in which the target genes were under the control of CaMV35s promoter. Then the recombinant vectors were transformed into the *Agrobacterium* strain GV3101, respectively. The transformed Agrobacterium strain was used to infect the WT *Arabidopsis thaliana* plants using floral dipping method [40]. Transgenic *Arabidopsis* were screened on 1/2 Murashige and Skoog (MS) agar media containing kanamycin. Flowering time was measured in the T3 generation using lines homozygous from several independent transformation events.

### Yeast two-hybrid assay (Y2H)

Both full-length ORF of *PvCO1* and *PvGF14c* were cloned into the pGBKT7 BD vector and pGADT7 AD vector for the swapping experiment. The truncated *PvCO1* fragments encoding the N-terminus region containing the two B-box domain (Met1-Leu150) was cloned into the pGBKT7 BD vector, and the other truncated *PvCO1* encoding the C-terminal (Met269-Phe384), was also cloned into the pGBKT7 BD vector. Both pGBKT7 BD vector and pGADT7 AD vectors were co-transformed into the yeast strain AH109. The positive transformants were selected on SD- Leu-Trp agar medium and then transferred to SD-Trp-Leu-His-Ade agar medium to identify the interaction in yeast. The positive and negative controls were from the kits cited below. The Y2H was performed according to the BD Matchmaker Library Construction & Screening Kits instructions (Clontech, Palo Alto, CA, US).

### Pull down

The full-length ORF of *PvCO1*, the truncated *PvCO1* fragments encoding the N-terminus region containing the two B-box domain (Met1-Leu150) and the other truncated *PvCO1* encoding the C-terminal (Met269-Phe384) were cloned into pET28a vectors (His tag), respectively. *PvGF14c* was cloned into pGEX-4 T-1 vector (GST tag). GST- *PvGF14c* and His-PvCO1 proteins were expressed in *E. coli* strain Rosseta and purified with glutathione sepharose 4B (GE Healthcare). Equal amounts of GST- PvGF14c protein coupled to glutathione sepharose 4B and His-PvCO1 proteins were incubated in PBS Buffer. The beads were then washed with PBS buffer. Bound proteins were eluted in elution buffer (50 mM Tris-HCl, 10 mM reduced glutathione, pH 8.0), separated by 12% SDS–PAGE, transferred to a polyvinylidene difluoride (PVDF) membrane (BIO-RAD, USA), and immunoblotted with anti-His antibody or anti- GST (Abmart, China). After washing the membranes with PBS buffer containing 0.2% Tween (PBST), the membranes were incubated for 1 h with anti-mouse IgG conjugated to horseradish peroxidase (Proteintech, USA). Detection was performed using Clarity Western Chemiluminescence (ECL) Substrate (BIO-RAD, USA) and visualized using a ChemiDoc MP system (BIO-RAD, USA).

### Statistical analyses

All statistical analyses were performed using SPSS v19.0 (SPSS Inc., Chicago, IL, US). The data from independent assays are analysed using analysis of variance (ANOVA, GLM procedure) and presented as the mean ± SD. Differences at *P* < 0.01 were considered highly significant.

## Results

### Cloning and sequence analysis of two *PvCOLs* in *Ph. violascens*

To identify the *CO* homologous gene in the bamboo species, *Ph. violascens*, we performed a local blastn and blastp search of rice *COL* sequences against the Moso Bamboo Genome Annotation database (http://202.127.18.221/bamboo/down.php). Fifteen *COL* genes belonging to the *COL* gene family were obtained in this 31,987 functional annotation database. However, another two genomic DNA sequences very similar to *Hd1* were screened only in draft genome sequence, and these two cDNA or protein sequences were not found in 31,987 functional annotation database. Therefore, we designed a pair of primers TOHLF1/TOHLR2 based on the conserved sequence of *CO* genes from rice, maize and wheat to generate these two *CO* homologous with genomic DNA as template from the bamboo species, *Ph. violascens*, using PCR amplification. Two DNA fragments (1,433 bp and 1,273 bp, respectively) were obtained with the pair of primers. Sequence analysis indicated that the two fragments showed high identity with rice *Hd1* (orthologous to *CO*), named *PvCO1* and *PvCO2*. We also performed blastn against the NCBI database, and obtained one *COL* (referred as *COL1*) sequence from *Ph. heterocycla* that had 98% identity with *PvCO1* fragment sequence using *PvCO1* as the query and another *COL* (named *COL2*) sequence from *Dendrocalamus xishuangbannaensis* that showed 92% identity with the *PvCO2* fragment sequence using *PvCO2* as the query.

**Fig. 1:**
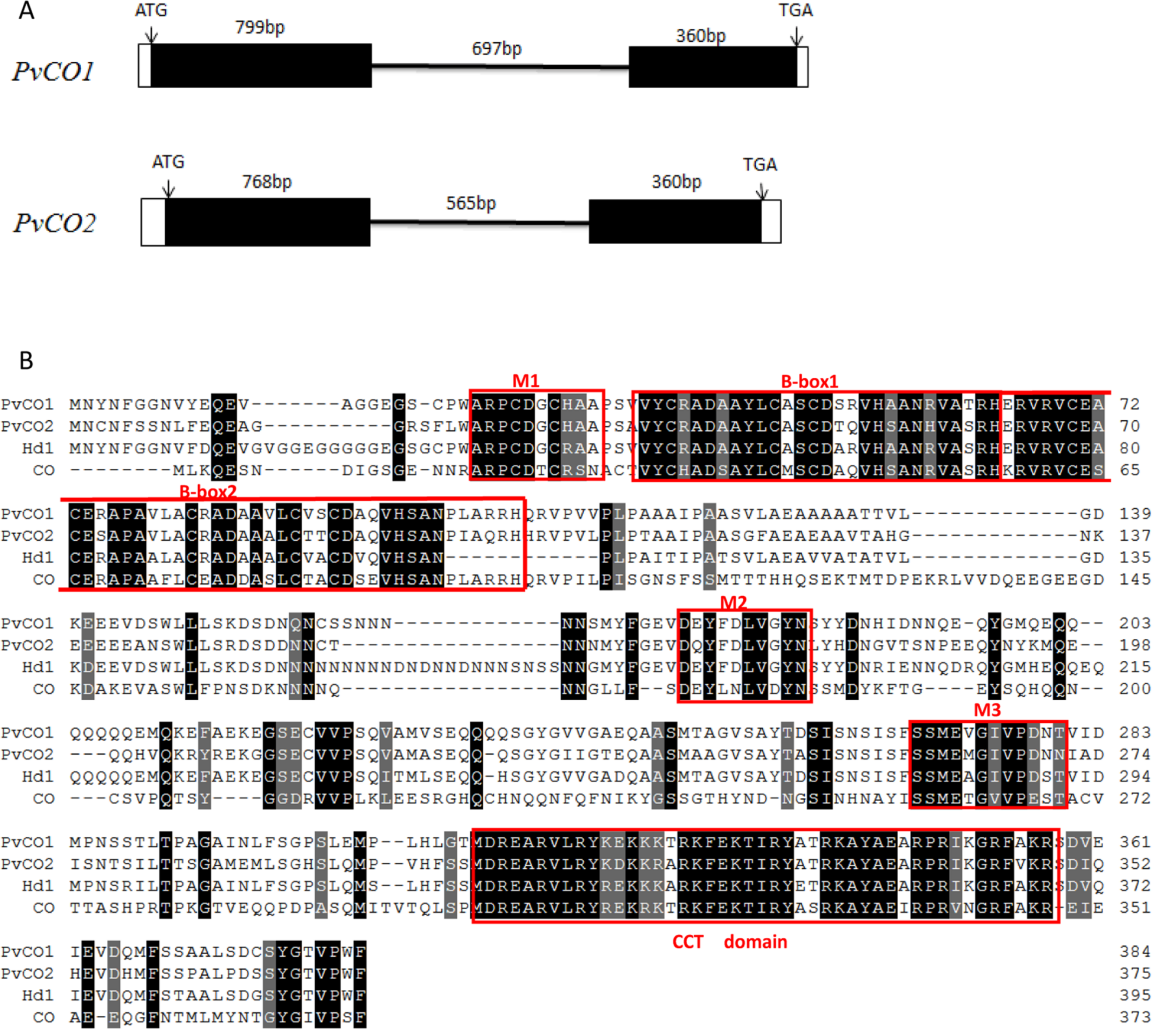
Bioinformatic analysis of *PvCO1* and *PvCO2.* **a** Genomic structures of *PvCO1* and *PvCO2.* The filled boxes indicated the exons, the black lines indicated introns, and the open boxes indicated upstream sequence or downstream sequence. Numbers represented the sizes of each region in base pairs. **b** Alignment of deduced amino acid sequences of PvCO1, PvCO2, Hd1 (rice) and CO (*Arabidopsis*). The conserved B-box and CCT domains of CO homologs are boxed. In red M1, M2, and M3 are additional conserved regions between the four CO homologs. Amino acids which are 100% conserved are shaded in black and partially conserved are shaded in gray

**Fig. 2:**
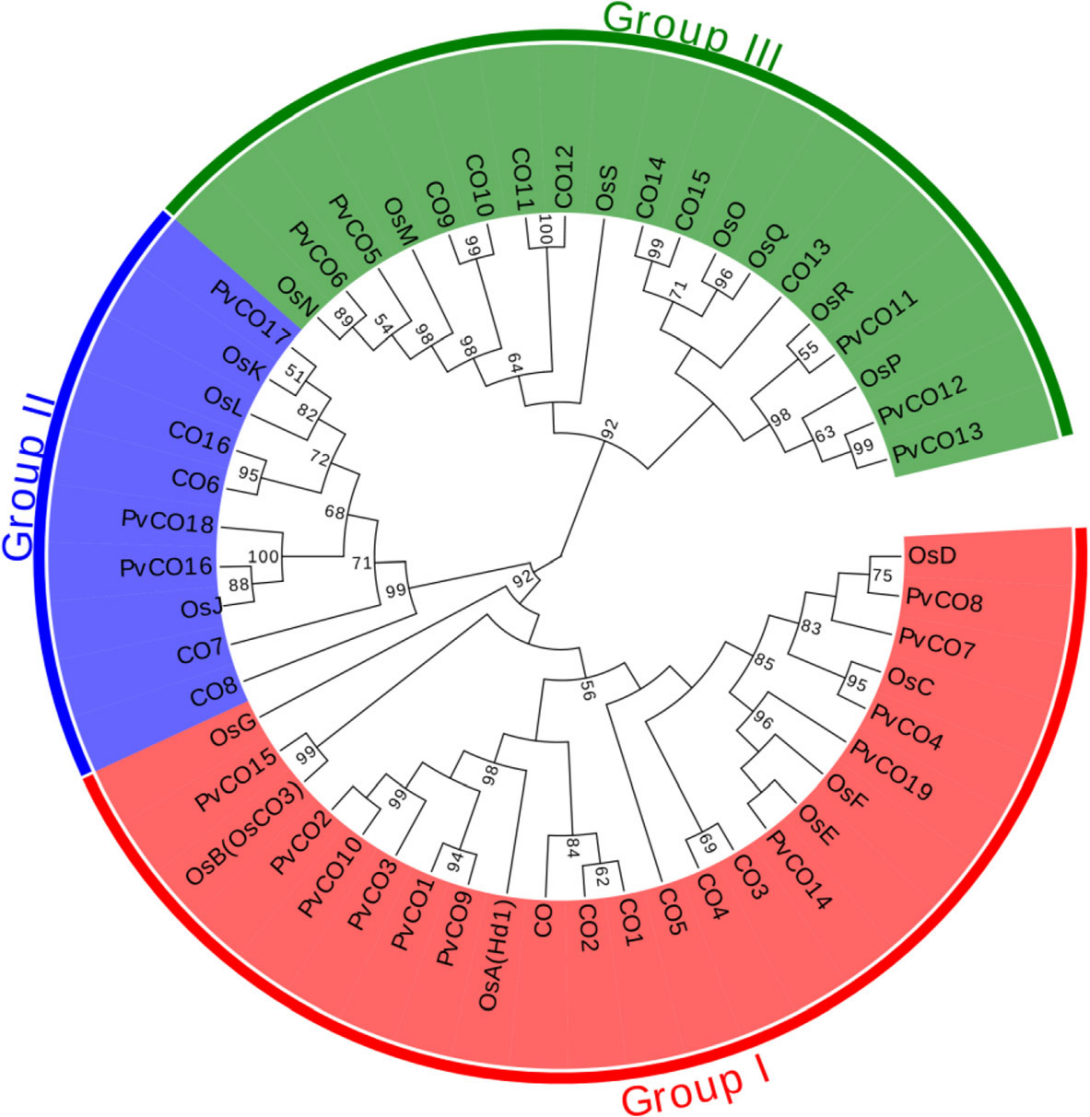
Phylogenetic tree of COL proteins in *Arabidopsis thaliana*, *Oryza sativa*, *Ph.violascens* and *Ph. heterocycla*. Bootstrap analysis (1000 replicates) was performed to assess the support of each branch. Accession numbers of the CO proteins used to construct the phylogenetic tree are listed in Additional file 3: Table S3

To obtain the complete *PvCO1* gene, we designed primers for genome walking and RACE based on the *COL1* sequence. Two fragments (850 bp and 550 bp-long, respectively) were amplified from the 5′ end and 3′ end, respectively. After analysis of the obtained sequences, we obtained a 2,535 bp DNA sequence by assembling the three fragments. Finally, a 1,942 bp fragment from genomic DNA and a 1,241 bp fragment from cDNA were amplified with primer PvCO1F and PvCO1R by end-to-end PCR. To obtain the full length sequence of *PvCO2* gene, we also designed a pair of primers PvCO2F and PvCO2R based on the *COL2* sequence. An 1,825 bp DNA sequence and a 1,260 bp cDNA sequence were amplified using the primers PvCO2F and PvCO2R, respectively. Analysis of the DNA and cDNA sequences suggested that both *PvCO1* and *PvCO2* genes had two exons and one intron (Fig. 1a). All of exon-intron junction followed the GT-AG rule. The ORFs of *PvCO1* (accession number: MH459145) and *PvCO2* (accession number: MH459146) were 1,155 bp and 1,128 bp-long and encoded 384 and 375 amino acids, respectively. Both PvCO1 and PvCO2 contained the conserved domain: two B-box domains, and a CCT domain (Fig. 1b). The PvCO1 protein showed 79.4% identity with Hd1 and 40.0% identity with CO, the PvCO2 protein 61.7% identity with Hd1 and 38.7% identity with CO. PvCO1 protein shared 70.9% identity with PvCO2. The alignment of PvCO1, PvCO2, Hd1 and CO indicated that besides the B-box and CCT conserved domains, three other small conserved regions, M1, M2 and M3, were identified although the other regions diverged (Fig. 1b). Phylogenetic analysis also showed that both PvCO1 and PvCO2 shared high identities with Hd1 (Fig. 2). The two PvCOL proteins clearly belonged to the COL gene family.

### *PhCOL* gene family members in *Ph. violascens*

In our previous study, the identities of homologous genes between *Ph. heterocycla* and *Ph. violascens* were found to be > 95%. Using *PvCO1* as a query for local BLASTn analyses against the Moso Bamboo Genome DNA database, we obtained several *COL* genes. Among them, one (named *PhCO1*) showed 98.25% identity with *PvCO1*. Another gene (named *PhCO2*), having 97.99% identity with *PvCO2*, was obtained using the same method. This suggested that *COL* corresponding homologous genes in *Ph. heterocycla* and *Ph. violascens* had a close relationship. Using rice *COL*, *PvCO1* and *PvCO2* as queries, we found 19 *COL* gene members in the *Ph. heterocycla* genome. All of them contained B-box domain and CCT domain. Then, according to these sequences, we designed primers (Additional file 2: Table S2) to amplify these 19 corresponding *COL* gene members from *Ph. violascens*. The 19 PvCOL proteins (Additional file 3: Table S3) were divided into three groups based on the identities of amino acid sequences similar to *Arabidopsis* and rice COL proteins (Fig. 2, Additional file 4: Table S4). In addition, based on variations within the B-box region, the 19 COL proteins group into three clusters: the first group (PvCO1, PvCO2, PvCO3, PvCO4, PvCO7, PvCO8, PvCO9, PvCO10, PvCO14, PvCO15, PvCO19) has two B-box domains, the second (PvCO5, PvCO6, PvCO11, PvCO12, PvCO13) has one B-box and a second diverged B-box, the third (PvCO16, PvCO17, PvCO18) has one B-box (Additional file 5: Figure S1). In group one, two homologous pairs, PvCO1/PvCO9 and PvCO2/PvCO3/PvCO10, had the highest sequence similarity to rice OsA/Hd1. Another homologous pair, PvCO14/PvCO19 was highly homologous to rice OsE. The fourth pair, PvCO8/PvCO7, had the closest relationship to the rice OsD. In the second group, there was one pair, PvCO12/PvCO13 with the highest identity to OsP. These data suggest that COL tandem duplication may have occurred in the genome of the bamboo species, *Ph. violascens*, during species evolution.

### Expression of *PvCO1* and *PvCO2* genes

**Fig. 3:**
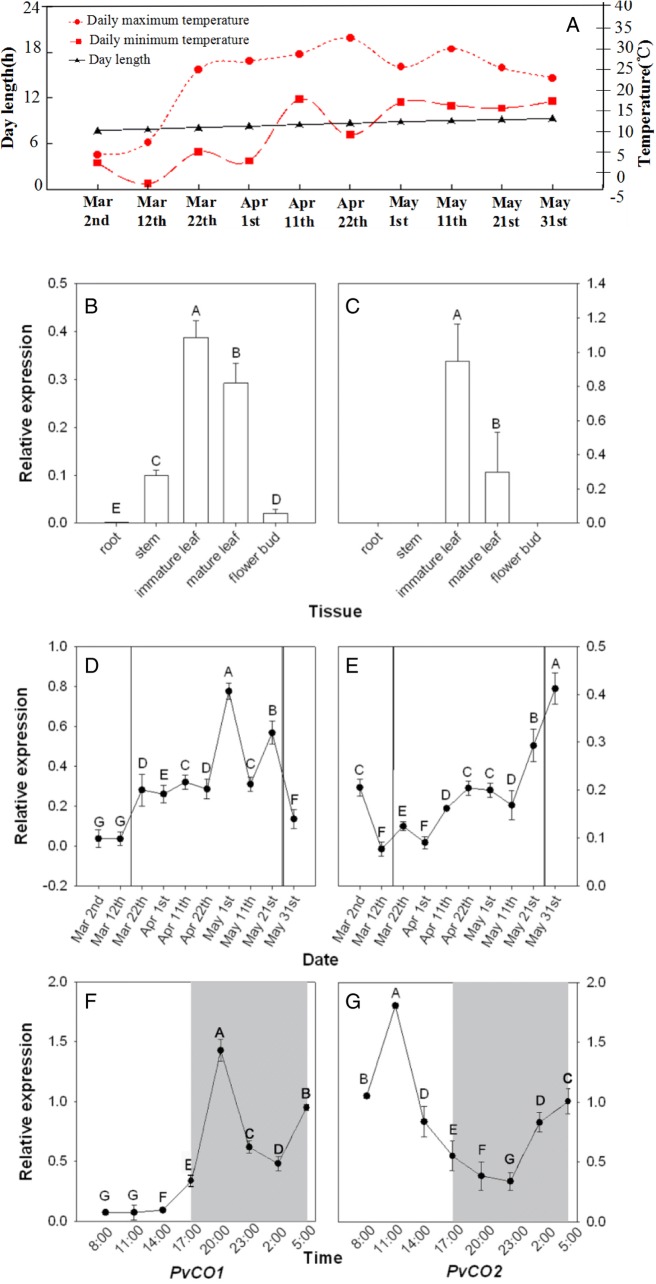
Expression analysis of *PvCO1* and *PvCO2*. **a** Daily high and low temperatures and daylength in lin’an (China). **b**, **c** Tissue-specific expression of *PvCO1* and *PvCO2* in *Ph. violascens*. The different tissues were collected from 5 pm to 6 pm on April 13 (LD 13:11 h). The maximum and minimum temperatures on April 13 were 17.3 °C and 13.9 °C, respectively. **d**, **e** Temporal expression of *PvCO1* and *PvCO2* in *Ph. violascens*. The three plants for samples flowered for the second time. The flower bud of these three plants displayed between on March 12 and 22, and they died between on May 21 and May 31. The period between black solid lines represents flowring time. **f**, **g** Daily expression of *PvCO1* and *PvCO2* in *Ph. violascens*. Dusk and dawn are at 17:00 and 5:30.Expression of the *Actin* gene was used as an internal control. Gray areas indicate darkness. Gray shading denotes the dark/night portion of 24 h cycle. Error bar indicates standard deviation. Y axis, relative transcript level of *PvCO1* or *PvCO2* compared with that of *PvActin*. A, B indicate highly significant differences

To study the expression level of the two target genes in different tissues, we conducted Reverse transcription PCR (RT-PCR) analysis and real-time PCR using total RNA isolated from the root, bamboo shoot, mature leaves, immature leaves, and flower buds. These tissues were from plants grown under natural conditions. The temperature and daylength were shown in Fig. 3a. The results showed that both *PvCOLs* were tissue specific in expression (Fig. 3b and c). *PvCO1* expression was detectable in immature leaves, mature leaves, and stems. It had greater abundance in immature leaves and mature leaves than in stems. *PvCO2* transcript was detected only in leaves, but not in the roots, stems, and flower buds. These data also demonstrated that transcript accumulation of the two target genes was very low and was mainly in the leaves.

To examine the temporal expression pattern of the two target genes, real-time PCR and RT-PCR analysis was performed using total RNA isolated from field-collected bamboo leaves. Fig. 3d and e showed that expression of both *PvCO* genes fluctuated. *PvCO1* mRNA level increased after March 12, maintained a high level during flowering from March 22 to May 21, and sharply declined to the initial level after May 21. This suggested that the transcript of the *PvCO1* gene was present during flowering period. *PvCO2* mRNA abundance was detectable at low levels during the entire period from March 2 to May 11, and then quickly increased.

The circadian expression of the *PvCO1* and *PvCO2* genes was confirmed under natural conditions using total RNA isolated from leaves collected at different times within one day. The samples were taken 3 h apart, starting at 8:00 am and ending at 5:00 am. The *PvCO1* gene expression level increased at dusk and maintained a high level throughout the night. However, the transcript accumulation of *PvCO2* was higher in the morning than at other times (Fig. 3f and g). These expression pattern results suggested that *PvCO1* might be associated with flowering in *Ph. violascens.*

### Overexpression of *PvCO1* delays the flowering time under LD conditions in *Arabidopsis*

**Fig. 4:**
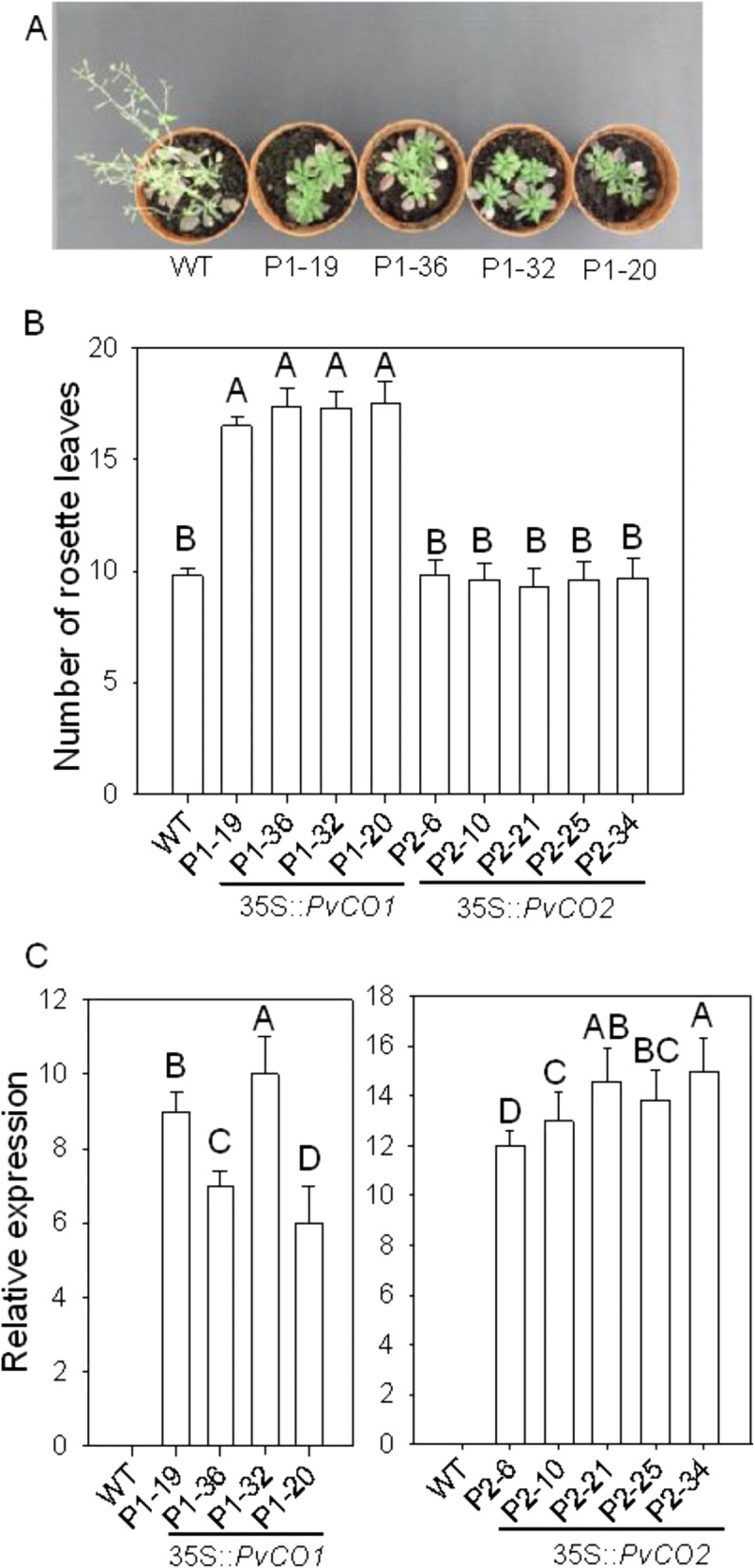
Phenotypes of 35S::PvCO1 or 35S::PvCO2 transgenic plants. **a** Flowering phenotypes of wild type and 35S::PvCO1 lines. **b** Analysis of number of rosette leaves of different genotype plants at blooming. **c** Real-time quantitative RT-PCR analysis of transcript accumulations of *PvCO* in different transgenic lines. The total RNAs were isolated from 14 d seedlings at ZT 4 h under LD.Y axis, relative transcript level of *PvCO1* or *PvCO2* compared with that of *AtActin*. Error bar indicates standard deviation. **a**, **b** indicate highly significant differences. At least 15 plants per line from the T3 generation were grown to analyze the flowering time phenotype

To investigate the effect of *PvCO1* and *PvCO2* genes on flowering time, we overexpressed *PvCO1* and *PvCO2*, under the control of the 35S promoter in WT *Arabidopsis* by *Agrobacterium*-mediated transformation, respectively. A total of 20 independent *PvCO1* transgenic plants and 8 independent *PvCO2* transgenic plants exhibiting kanamycin resistance in the T1 were confirmed by PCR. Four *PvCO1* transgenic lines and five *PvCO2* transgenic lines from the T3 generation were selected to study the flowering time of transgenic *Arabidopsis* plants. All four *PvCO1* transgenic lines flowered significantly later than wild-type *Arabidopsis* (Fig. 4a). In contrast, the flowering time of five *PvCO2* transgenic lines showed no difference with that of WT *Arabidopsis* (data not shown). At least 15 plants per line from the T3 generation were grown to analyze the flowering time phenotype. The time of flowering was determined by counting the number of rosette leaves when floral buds were first visible.

As shown in Fig. 4b, *PvCO1* transgenic lines flowered significantly later 10–15 d than WT plants. The *PvCO2* transgenic line was similar to WT *Arabidopsis. PvCOLs* transcript levels were studied in WT *Arabidopsis* and several independent transgenic lines overexpressing *PvCO1* or *PvCO2* using the total RNA isolated from 14 d seedlings at ZT 4 h under LD. As expected, compared to WT, expression levels of *PvCOLs* in different transgenic lines were significantly increased. However, there was no correlation between the *PvCOLs* mRNA abundance and the flowering time (Fig. 4c and d). These results indicated that *PvCO1* repressed flowering times in *Arabidopsis* under LD conditions and this might suggest the possibility that *PvCO1* represses flowering in *Ph. violascens.*

### *PvCO1* negatively affect *AtFT* expression

**Fig. 5:**
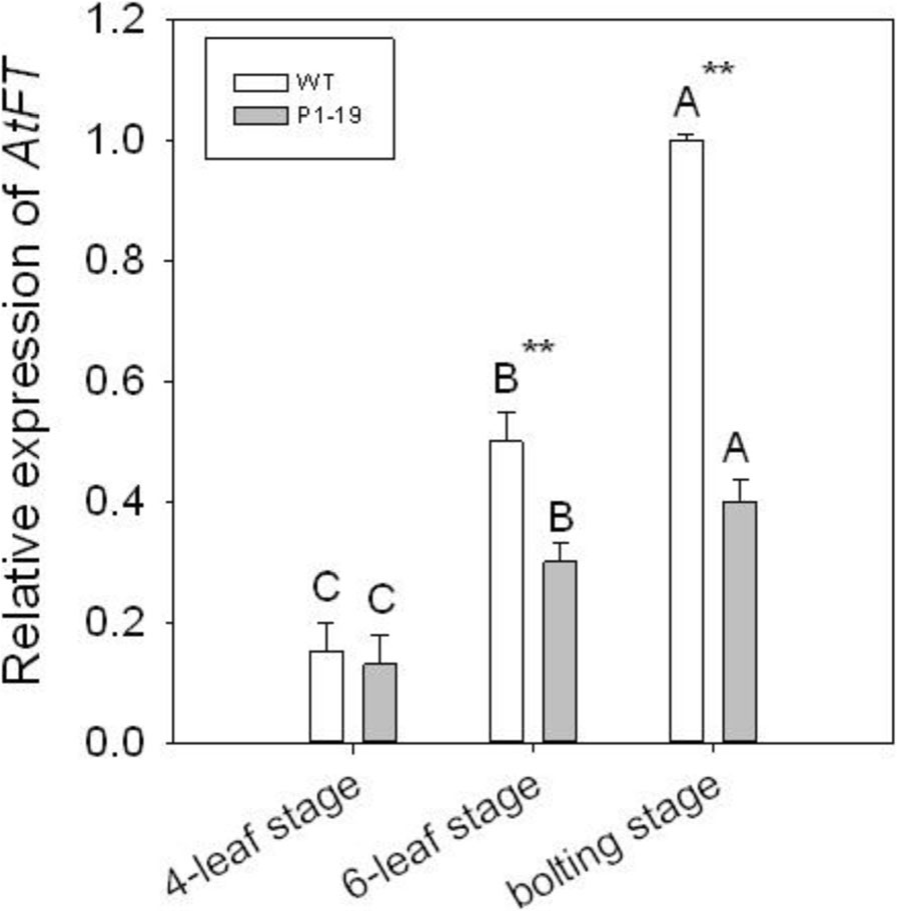
Expression of *AtFT* in *PvCO1* transgenic lines. Leaf were sampled from plants in the 6-leaf and bolting stages under LD, respectively. Y axis, relative transcript level of *AtFT* compared with that of *Arabidopsis* actin. Error bars indicate standarddeviations. A, B indicate highly significant differences. The asterisk denotes a statistically significant difference between WT and transgenic line P1–19 (*P* < 0.01). *n* = 4 or more

To study the molecular mechanisms by which *PvCO1* controls flowering time in *Arabidopsis*, we analyzed the expression level of *AtFT* in WT, and transformed lines (P1–19) at three developmental stages (4 leaf, 6-leaf, and bolting). The results showed that *AtFT* transcript levels in p35S::*PvCO1* lines were lower than those in WT in the 6-leaf and bolting stages (Fig. 5). This suggests that *PvCO1* can reduce the *AtFT* expression level and thus delay flowering in transgenic *Arabidopsis.*

### PvCO1 can interact with 14–3-3(PvGF14c) protein

**Fig. 6:**
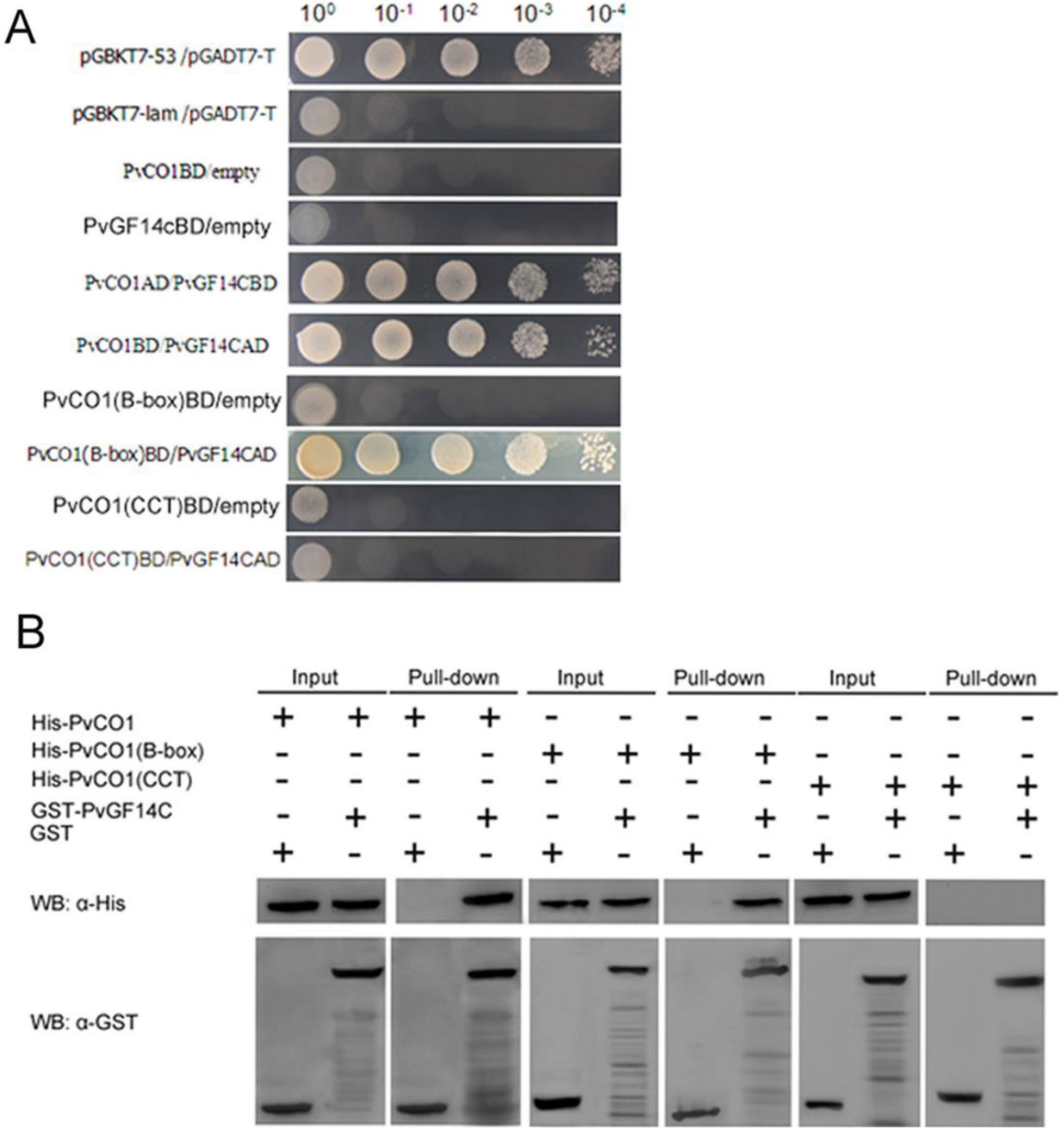
The PvCO1-PvGF14c interaction by yeast two-hybrid (**a**) and pull down (**b**) assays. **a** The pGBKT7–53 and pGADT7-T interaction was used as a positive control. The pGBKT7-lam and pGADT7-T interaction was used as a negative control. The PvCO1 and empty interaction was used as self-activation test. PvCO1 indicated full length ORF of *PvCO1* gene. PvCO1(B-box) indicated the truncated CO protein containing the two B-box domain. PvCO1(CCT) indicated the truncated CO protein containing the CCT domain. **b** The addition of GST or GST-PvGF14c is indicated in each line. The antibodies used for Western blotting are abbreviated as WB:α-His and WB:α- GST. Input, the proteins from whole cell extracts; Pull-down, the pull down proteins

In *Arabidopsis*, CO protein can interact with 14–3-3 isoforms affecting photoperiod controlled flowering [28]. Our studies confirmed that overexpression of 14–3-3 protein c (PvGF14c), one of 14–3-3 gene family in *Ph. violascens*, delayed flowering time in *Arabidopsis* [41]. To verify whether PvCO1 can interact with PvGF14c protein, yeast two-hybrid screening and pull down were performed. Fig. 6a showed that PvCO1 and PvGF14c could not be self-activated. But PvCO1could interact with PvGF14c protein either as bait or as prey. Previous studies indicated that the B-box domain or CCT domain can be involved in protein-protein interactions. To determine which conserved domain was sufficient for the interaction between PvCO1 and PvGF14c, we constructed truncated PvCO1 protein that only possessed two B-box domains (PvCO1(B-box)) or CCT domain (PvCO1(CCT)) to interact with the PvGF14c protein. As shown in Fig. 6a, PvCO1(B-box) and PvCO1(CCT) could not be self-activated. PvGF14c interacted with the PvCO1(B-box) and did not interact with the PvCO1(CCT). These results indicate that two B-box domain of PvCO1 was sufficient for interaction with PvGF14c and CCT domain was not sufficient to interact with PvGF14c. Pull down assays further confirmed a strong binding between PvCO1 and PvGF14c through by B-box domains not by CCT domain (Fig. 6b).

### Both PvCO1 and PvCO2 localize to nucleus

**Fig. 7:**
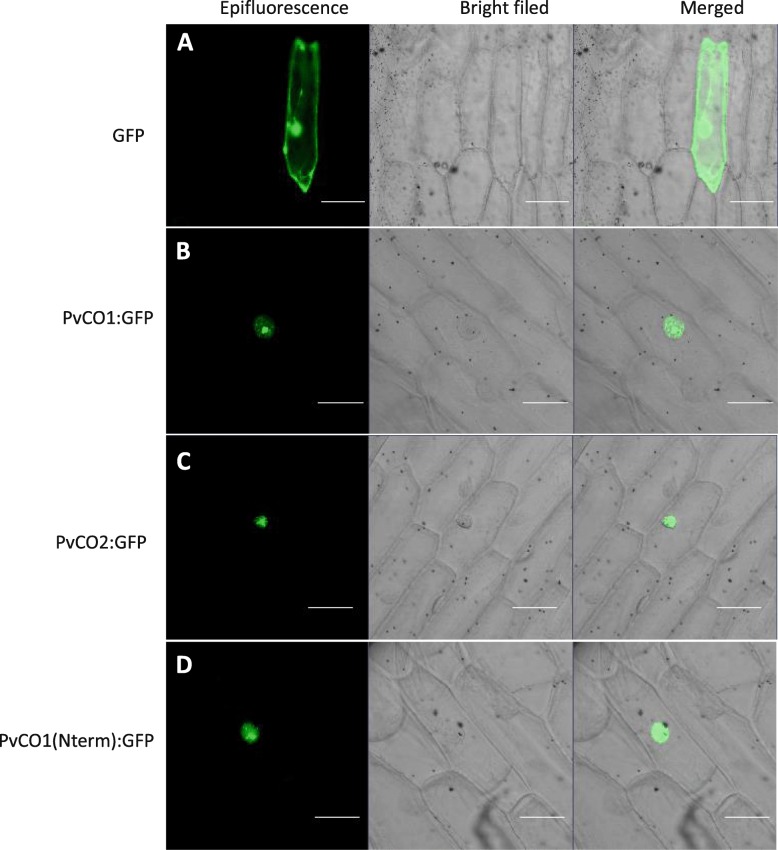
Subcellular localization of PvCO1 and PvCO2. **a** Control GFP plasmid. **b** Subcellular localization of PvCO1:GFP fusion protein. **c** Subcellular localization of PvCO2:GFP fusion protein. **d** Subcellular localization of PvCO1(Nterm):GFP fusion. The left verticals are green fluorescence images, middle verticals are bright-filed images, and right verticals are merged images of bright field and green fluorescence. Scale bars in this figure are 100 μm

To assess the molecular function of PvCO1 and PvCO2, we made PvCO1 or PvCO2 protein-linked GFP fusion constructs driven by the CaMV35S promoter and used these to analyze the intracellular localization of PvCO1 and PvCO2. To understand the role of the CCT domain in PvCO1, we constructed the CCT domain peptide-linked GFP fusion vector driven by the CaMV35S promoter to analyze the intracellular localization of the CCT domain. These constructs were introduced into onion epidermal cells for transient expression. The empty GFP signals were ubiquitously distributed throughout the cells (Fig. 7a). Both PvCO1-GFP fusion protein and PvCO2-GFP fusion protein were only present in cell nuclei (Fig. 7b and c). These results confirmed that PvCO1 and PvCO2 are nuclear-localized protein. The PvCO1 (Nterm)-GFP fusion protein that contained the CCT domain of PvCO1 was also observed only in cell nuclei (Fig. 7d). These data suggest that the CCT domain of PvCO1 may act as a nuclear localization signal.

## Discussion

Bamboo grown under natural conditions has a wide range of flowering times. Some species have a lengthy vegetative stage that may last more than 120 years while other species flower after only 1 year. Little is known about floral induction in bamboo or the genes involved in the process [6, 15–17, 42].

We identified and characterized two genes, *PvCO1* and *PvCO2* in the bamboo *Ph. violascens*. These genes are homologs of the *CO* in *Arabidopsis* and *Hd1* in rice. Both *PvCOL* genes consisted of two exons and one intron (Fig. 1a). They shared two B-box domains containing typical zinc finger structures near the N-terminal and a conserved CCT domain near the C-terminal (Fig. 1b), suggesting that both encode transcriptional factors. The predicted protein sequences of PvCO1 and PvCO2 had low similarity with CO, but the two B-box domains and the CCT domain were highly conserved and they showed high similarity to Hd1. The identity between PvCO1 and PvCO2 was 70.91%. The alignment of both PvCOLs, Hd1, and CO indicates that the COL protein family has been conserved in bamboo (Fig. 1b, Additional file 5: Figure S1).

*CO* belongs to a gene family composed of 17 *COL* genes in *Arabidopsis* [22]. There are similar numbers of *COL* genes in the genomes of rice, sorghum and foxtail millet [38]. A total of 19 *COL* genes were identified in *Ph. violascens*, indicating that the *CO* gene family in bamboo also has many members. Based on the number and variation of the B-box, these 19 PvCOL proteins were classified into three groups. Group I contains two B-boxes, group II has a B-box and a second diverged B-box, and group III contains one B-box (Additional file 5: Figure S1). The second diverged B-box lacks C or H residues, or has a substitution of the conserved C or H residue. However, the CCT domain of these COL proteins shows high similarity among rice, sorghum, foxtail millet, and bamboo. Excluding the B-box and CCT regions, the remaining regions had high variation in the COL proteins among the four species. Phylogenetic analysis of the COL gene family in *Arabidopsis*, rice and bamboo also resulted in three groups (Fig. 2). All of the *COL* genes in *Ph. violascens* had corresponding genes in rice. In every group, there was a gene pair in bamboo corresponding to a single gene in rice. For example, PhCO14/ PhCO19 (92.49% identity) had highest homologies to rice OsE. Group I includes most of the genes known to have COL function in other species and contains 11 of the 19 PvCOLs. These results correspond with the multiple genome duplication events that have occurred in bamboo. Analysis of single-copy genes and gene families that contained 2–4 gene members show fewer single-member gene families and more two-member families in the *Ph. heterocycla* genome than in the rice genome [4]. Collinearity investigation of orthologous genes between bamboo and rice indicated that a whole-genome duplication event occurred in bamboo [4]. Similar to maize, there may have been a tetraploidization event(s) during bamboo evolution [4]. In rice, most of the seventeen COL genes form paralogous gene pairs (OsC-OsD, OsE-OsF, OsK-OsL, OsM-OsN, OsO-OsQ, OsP-OsR) [38]. However, phylogenetic trees showed that COL genes in *Ph. violascens* had greater homology compared to gene pairs from rice. This may indicate that the divergence of gene pairs of COL predated the bamboo/rice divergence.

In most flowering plants, the activity of *CO* and its orthologous genes are regulated by photoperiod and shows a circadian rhythm that varies among different species. In *Arabidopsis*, CO expression, and control of flowering, is regulated by the circadian clock [19, 20]. CO expression was modulated by the circadian clock and day length and it peaked twice (dawn and dusk) under LD conditions [20, 43] and accumulated mostly during the dark period under SD conditions [43]. In rice, the *Hd1* mRNA level was low at midday and highest during the night regardless of LD or SD conditions [44]. Tomatoes (*Lycopersicum*) are a day-neutral species and the effect of day length on peak expression time of the *TCOL1* and *TCOL3* genes was similar to that of *CO* in *Arabidopsis* [45]. In *Populus trichocarpa*, *PtCO2* expressed in a diurnal pattern, peaking at the end of the day under LD conditions and having a low expression peak at night under SD conditions [35]. In *Ph. violascens*, *PvCO1* displayed a diurnal pattern with higher expression during the night than the day under a LD 12.5:11.5 h photoperiod. This diurnal expression pattern was not completely consistent with, but similar to the expression pattern of other *CO* orthologous genes, suggesting that light and the circadian clock modulated the peak of *PvCO1*. Our expression studies also show that *PvCO1* mRNA abundance accumulated from 5:00 pm to 5:00 am, unlike the high expression of *PvFT1* from 2:30 pm to 8:30 pm [6]. The time lag between the expression of *PvCO1* and *PvFT1* suggests the possibility that the *CO/FT* regulatory module is not strongly conserved and that there are unidentified mechanisms necessary for *PvFT1* induction in *Ph. violascence.* CO and its orthologous gene regulated flowering time at a low level of expression. They were detected only by RT-PCR and were not found in the libraries screened [21, 29]. Their transcripts were not tissue-specific and were present in most of tissues examined [21, 29, 46–50]. Corresponding cDNA sequences of the two *PhCOL* genes in moso bamboo, *PhCO1* and *PhCO2*, were not found in the 31,987 protein-coding gene database (http://202.127.18.221/bamboo/), and both *PvCOL* genes were detected only by RT-PCR, demonstrating that their mRNA accumulation was very low. Despite high sequence similarity between *PvCO1* and *PvCO2* which had highest identity to OsA/Hd1, they showed highly diverse expression patterns. The transcripts of *PvCO1* were detected in leaves and stems, exhibited higher expression during the flowering stage, than stages before and after flowering, and showed a daily pattern under natural conditions with higher expression in darkness than in daylight, similar to *CO*/*Hd1* of *Arabidopsis* and rice. In contrast, *PvCO2* was only found in leaves and very low during the flowering stages, its expression levels were higher in the morning than at other times under the same conditions. These data suggested that *PvCO1* and *PvCO2* genes were functional differentiation and *PvCO1* had more relevance to the flowering process while *PvCO2* might be a paralog of *CO*/*Hd1*.

Based on the amino acid sequence, COLs belong to the zinc finger gene family and act as transcription factors [21, 22, 51, 52]. They are localized in the nucleus and bind the promoter region of downstream target gene *FT* to activate *FT* transcription by itself or via complex formation [24, 27]. Either the B-box domain or CCT domain can interact with the other protein and then induce *FT* transcription [53]. Song et al. [54] reported that through the B-box domain, the CO protein can partially regulate *FT* transcription by forming a complex with ASYMMETRIC LEAVES 1 (AS1) protein. The B-box domain can also interact with the TGA4 transcription factor [22, 54]. Through the CCT domain, CO interacts with HEME ACTIVATOR PROTEIN (HAP) trimetric transcription factor complex, which regulates *FT* expression [27], as well as the COP1-SPAs E3 ubiquitin ligase complex, and then stabilizes CO protein [55, 56]. CCT may also function as a nuclear-localization signal for protein transport [21, 22]. In addition, previous reports suggest that the 14–3-3 proteins μ and υ influence the flowering transition and can interact with CO protein in *Arabidopsis* [28]*.* Our studies confirmed that PvCO1 was localized in the nucleus via CCT domain, and PvCO2 was also localized in the nucleus. PvCO1 could interact with 14–3-3 protein c (PvGF14c) through the B-box domain but not the CCT domain (Fig. 6). In the plant, 14–3-3 proteins could influence their binding partners at the spatiotemporal and subcellular levels as well as post-translational modification and stability [57]. Whether the interaction between PvCO1 and PvGF14c could affect the PvCO1 stability and nuclear transport need ongoing studies.

In other plant species, the expression and function of *CO* genes may be less conserved. CO can act as a inducer of floral transition in *Arabidopsis*, rice, potato, tomato, soybean and sugar beet [29, 34, 43, 58–61]. It is unclear the extent to which CO function has been preserved in poplar. Bo¨hlenius et al. [35] reported that the CO2/FT1 regulon controls the onset of reproduction in poplar, whereas, Hsu et al. [62] indicated that overexpression of CO1 and CO2 singly or together did not alter normal reproductive onset of poplar. In long-term field trials, overexpression of CO1 was able to complement the *Arabidopsis co*-2 mutant under long days. None of the eight *MtCOL* genes in *Medicago truncatula* could rescue the late-flowering phenotype of *co Arabidopsis* [63]. In contrast, the group I genes *CO3*, *OsCO3*/*OsB* and *OsCOL4*/*OsD*, group II gene *OsCOL10, OsCOL13, OsCOL16,* as well as the group III gene *AtCOL9* inhibited flowering [64–69]. Our data showed that PvCO1, but not PvCO2, regulated the flowering time by reducing the expression of *FT* in *Arabidopsis*, because overexpression of PvCO1 caused floral delay, and overexpression of PvCO2 had no influence on the flowering time of *Arabidopsis* under long day conditions. Phylogenetic analysis showed that PvCO1 and PvCO2 clustered together with *Arabidopsis* CO and rice Hd1. This suggests the possibility of PvCO2 evolving a novel function, having no role in flowering regulation, or acting redundantly with other flowering regulators in *Arabidopsis.* The results indicate that the functions of CO in regulating flowering time are complex and diverse. It is likely that the long period of bamboo vegetative growth is related to the flowering inhibition regulator of PvCO1.

## Conclusion

Two *COL* genes, *PvCO1* and *PvCO2*, from *Ph. violascens* were identified. Both genes had different expression patterns. The expression of *PvCO1* was related to floral transition, but expression of *PvCO2* was not. Levels of both *PvCO1* and *PvCO2* mRNA displayed a circadian pattern. Overexpression of *PvCO1* delayed flowering in *Arabidopsis,* while overexpression of *PvCO2* has no effect on *Arabidopsis* flowering time. The long period of vegetative growth of bamboo may be related to an inhibition regulator of *PvCO1*.

## Additional files

Additional file 1: **Table S1.** Primers used for cloning *PvCO1* and *PvCO2* genes. (DOCX 24 kb)

Additional file 2: **Table S2.** Primers used for cloning 17 *PvCO* genes in *Ph. violascens*. (DOCX 20 kb)

Additional file 3: **Table S3.** Accession numbers of *COL* gene family members in *Arabidopsis*, *Oryza sativa* and *Ph. heterocycla*. (DOCX 23 kb)

Additional file 4: **Table S4.** Characterization of *COL* gene family members in *Ph. violascens*. (DOCX 23 kb)

Additional file 5: **Figure S1.** Alignment of predicted N and C amino acid sequences of partial COLs from *Arabidopsis thaliana, Oryza sativa, Ph. violascens,* and *Ph. heterocycla*. The B-box domain and CCT domain are labeled by red lines. The second diverged B-box in group II is labeled by red box. (DOCX 117 kb)

## Abbreviations

*AP1*: *APETALA1*
CO: CONSTANS
FT: Flowering locus T
Hd3a: Heading date 3a
LD: Long day
ORF: Open reading frame
RACE: Rapid amplification of cDNA end
RT-PCR: Reverse transcription PCR
SD: Short-day
