# Supplementary material for: Overexpression of PvCO1, a bamboo CONSTANS-LIKE gene, delays flowering by reducing expression of the FT gene in transgenic Arabidopsis
Source: BMC Plant Biol. 2018 Oct 12;18:232. doi: 10.1186/s12870-018-1469-0 (PMC6186071; doi:10.1186/s12870-018-1469-0)
Supplement: Supplementary file 1 — Table S1. Primers used for cloning PvCO1 and PvCO2 genes. (DOCX 24 kb) [file 12870_2018_1469_MOESM1_ESM.docx]

Table S1 Primers used for cloning *PvCO1* and *PvCO2* genes

| Primer name | Sequence(5’-3’) |
| --- | --- |
| Cloning primers | |
| TOHLF1 | GAGGAA/GGTGGACTCTTGG |
| TOHLR2 | TCAGAACCATGGAACAGTA |
| 5SP1 | TGGAGGATGGTGTTTCACTTGAAG |
| 5SP2 | TGGAGGATGGTGTTTCACTTGAAG |
| 5SP3 | CGATGTGGTTGTCGTAGTACGA |
| 3SP1 | TGACACCTGCAATCTCCTTATG |
| 3SP2 | ACCTGCTGGAGCCATCAATC |
| 3SP3 | CGTTATGCGACAAGGAAGGC |
| GSP1 | TCATCAATGGAGGTGGGTATAGTA |
| GSP2 | TATGCGACAAGGAAGGCGTACGC |
| GSP3 | ACGTTGGTGCCTCCTGGCGA |
| GSP4 | ATCACAGGAGACGCACAGCACTGC |
| PvCO1F | TACTACTACCTTCCCTGAGAC |
| PvCO1R | TGTCTCATTGATCCCATCAG |
| PvCO2F | AATAGGACACACTAGAKASTAGC |
| PvCO2R | CCATGTGTCTGTCAACAYAWT |
| Semi-quantitative primers | |
| PvCO1expF | ACAACAACAGCATGTACTTCGGTG |
| PvCO1expR | TCTTCTCCTTGTACCTGAGGACC |
| PvCO2expF | ATTGTACCAACAACAACATG |
| PvCO2expR | CATACCTTATGGTCTTCTCA |
| ActinF | GAGCGAGAAATTGTCAGGGA |
| ActinR | GATGGCTGGAAGAGGACCT |
| Real-time PCR primers | |
| PvCO1qexpF | CATTCCGGCTGCTTCTGTACTTG  -3 |
| PvCO1qexpR | TTGCATCCCATACTGCTCTTGGT |
| PvCO2qexpF | GTGCCTGTGCTACCACTCCCAACT  -3 |
| PvCO2qexpR | TCTGCTGAGGAGCCAAGAGTTCG  -3 |
| AtFTF | TGGTGGAGAAGACCTCAGGAACT |
| AtFTR | CTGCCAAGCTGTCGAAACAATAT |
| AtActinF | AAAACCACTTACAGAGTTCGTTCG |
| AtActinR | GTTGAACGGAAGGGATTGAGAGT |
| PvActinqexpF | CCCGGTGGTCTCTATGCCT |
| PvActinqexpR | TTGTTGCGCAGTATCTTGATTCG |
| Over-expression primers | |
| 35S-PvCO1F | TACGGATCCATGAATTATAATTTCGGT |
| 35S-PvCO1R | TACGTCGACTCAGAACCATGGAACAGT |
| 35S-PvCO2F | TACGGTACCCCAGTTGAAAGGCTTATAC |
| 35S-PvCO2R | TACTCTAGATCAGAACCATGGAACAGTA |
| [Subcellular](app:ds:subcellular)[localization](app:ds:localization) primers | |
| PvCO1(ORF)F | TTCGGGAGCTCATGAATTATAATTTCGG |
| PvCO1(ORF)R | AATGCTCTAGA GAACCATGGAACAGTAC |
| PvCO1F(B-box) | TTCGGGAGCTCATGAATTATAATTCGG |
| PvCO1R(B-box) | AATGCTCTAGAGCTGTTGCTGATGGA |
| PvCO1F(CCT) | TTCGGGAGCTCATATCTTTCTCATC |
| PvCO1R(CCT) | AATGCTCTAGA GAACCATGGAACAGTAC |
| PvCO2(ORF)F | TTCGGGAGCTCATGAATTGTAATTTCAG |
| PvCO2(ORF)R | AATGCTCTAGAGAACCATGGAACAGTACC |
| Two-Hybrid System primers | |
| PvCO1(ORF)THSF | TACGAATTCATGAATTATAATTTCGGT  )  R 5- TACGGATCCTCAGAACCATGGAACAGT-3BamHI |
| PvCO1(ORF)THSR | TACGGATCCTCAGAACCATGGAACAGT |
| PvCO1(B-box)THSF | TACGAATTCATGAATTATAATTTCGGT |
| PvCO1(B-box)THSR | TACGGATCCGCTGTTGCTGATGGA |
| PvCO1(CCT) THSF | TACGAATTCATATCTTTCTCATC |
| PvCO1(CCT) THSR | TACGGATCCTCAGAACCATGGAACAGT |
| PvGF14cTHSF | AAACATATGATGTCGCGGGAGGAGAATGT  R5- AAAGGATCCAGCACAGCGGCATCTCAAAG -3BamHI |
| PvGF14cTHR | AAAGGATCCAGCACAGCGGCATCTCAAAG |
| Pull down primers | |
| PvCO1(ORF)PDF | TACGAATTCATGAATTATAATTTCGGT  )  R 5- TACGGATCCTCAGAACCATGGAACAGT-3BamHI |
| PvCO1(ORF) PDR | TACGGATCCTCAGAACCATGGAACAGT |
| PvCO1(B-box) PDF | TACGAATTCATGAATTATAATTTCGGT |
| PvCO1(B-box) PDR | TACGGATCCGCTGTTGCTGATGGA |
| PvCO1(CCT) PDF | TACGAATTCATATCTTTCTCATC |
| PvCO1(CCT) PDR | TACGGATCCTCAGAACCATGGAACAGT |
| PvGF14cPDF | AAAGGATCCATGTCGCGGGAGGAGAATGT  R5- AAAGGATCCAGCACAGCGGCATCTCAAAG -3BamHI |
| PvGF14cPDR | TTTGTCGACAGCACAGCGGCATCTCAAAG |
