# Supplementary material for: Overexpression of PvCO1, a bamboo CONSTANS-LIKE gene, delays flowering by reducing expression of the FT gene in transgenic Arabidopsis
Source: BMC Plant Biol. 2018 Oct 12;18:232. doi: 10.1186/s12870-018-1469-0 (PMC6186071; doi:10.1186/s12870-018-1469-0)
Supplement: Supplementary file 2 — Table S2. Primers used for cloning 17 PvCO genes in Ph. violascens. (DOCX 20 kb) [file 12870_2018_1469_MOESM2_ESM.docx]

Table S2 Primers used for cloning 17 *PvCO* genes in *Ph. violascens*

| Gene name | Primer name | Sequence(5’-3’) |
| --- | --- | --- |
| PvCO3 | PhCO3F1 | GTGGCAATATGCAAGCACACA |
|  | PhCO3R3 | CCTGCCAGCCTTAGCAGTTC |
| PvCO4 | PhCO4F | CCCACAGAGCTCTAATCCGC |
|  | PhCO4R | CTTGGAAGTCCGGATCGCTT |
| PvCO5 | PhCO5F1 | TAGCTGACGCAAGACGTTGG |
|  | PhCO5R2 | TGTGCCAGTCGCAATAAGCA |
| PvCO6 | PhCO6F1 | TGTCAAAGTACTGTGGGCCG |
|  | PhCO6R2 | GCAGAAGCAGTTACGGTTGC |
| PvCO7 | PhCO7F | AAGCGCCAAAATTACAGCGG |
|  | PHCO7R | AGCGACGGTTCCTCAAACAA |
| PvCO8 | PHCO8F | AGAAGGCCAGAAACAGCGAG |
|  | PHCO8R | GTACAAGTCGGGATTGGGCA |
| PvCO9 | PhCO9F | CCGTTTACGAGCAGGAGGTT |
|  | PhCO9R | TCACTCTGGTAAGTCCATGTGC |
| PvCO10 | PhCO10F1 | AAGTGTGCGAACTCCGTAG |
|  | PhCO10R1 | GCTTGATGTATGTGTGGCG |
|  | PhCO10F2 | GAAAGTTCAAGGGCGATGT |
|  | PhCO10R2 | CTTGTCAGTTTCCTGTCCG |
| PvCO11 | PhCO11F1 | CCCTCTTGTCTCTTCTGCCG |
|  | PHCO11R2 | GGCCCATGTATGCTTCCTCAT |
| PvCO12 | PhCO12F1 | CAGTCGTTGGATCGCGTACT |
|  | PHCO12R2 | CGCCCATTGATCCTCAACCT |
| PvCO13 | PhCO13F1 | TTGGTGTGCTAGCGACTGAG |
|  | PHCO13R2 | TGCACTGAAACCATGCAAAACA |
| PvCO14 | PHCO14F | CCAAAAAGCCAACCCATCCG |
|  | PHCO14R | CCGTAGCCGAAGTCGAACAT |
| PvCO15 | PHCO15F1 | CCACCGGAGTTCTATGTGGC |
|  | PHCO15R2 | CATGGAAGGAACGCTCAGGT |
| PvCO16 | PhCO16F | CGAGGTGTTCGACGGAATGA |
|  | PHCO16R | GGGGGCGTCCTAATTCACAA |
| PvCO17 | PhCO17F | GTGGCGTGTTGCCATTTGAT |
|  | PhCO17R | AAGCAGCTTCGACGAGTTGT |
| PvCO18 | PhCO18F | AGCGAAGTGTTCGACGGAAT |
|  | PhCO18R | CGCTCTCAAAAACGGAAGCG |
| PvCO19 | PhCO19F | GAGACGCGTGTGATGATGGA |
|  | PhCO19R | CTCGTGGCGGCTACTTCAAT |
