# Supplementary material for: Overexpression of PvCO1, a bamboo CONSTANS-LIKE gene, delays flowering by reducing expression of the FT gene in transgenic Arabidopsis
Source: BMC Plant Biol. 2018 Oct 12;18:232. doi: 10.1186/s12870-018-1469-0 (PMC6186071; doi:10.1186/s12870-018-1469-0)
Supplement: Supplementary file 3 — Table S3. Accession numbers of COL gene family members in Arabidopsis, Oryza sativa and Ph. heterocycla. (DOCX 23 kb) [file 12870_2018_1469_MOESM3_ESM.docx]

Table S3 Characterization of *COL* gene family members in *Ph. violascens*

| **Gene name** | **Genomic (bp)** | **cDNA(bp)** | **Exons** | **Protein (aa)** | **Protein domain** |
| --- | --- | --- | --- | --- | --- |
| PvCO1 | 1856 | 1155 | 2 | 380 | B1,B2,CCT |
| PvCO2 | 1693 | 1128 | 2 | 375 | B1,B2,CCT |
| PvCO3 | 6178 | 1992 | 6 | 663 | B1,B2,CCT |
| PvCO4 | 1098 | 999 | 2 | 332 | B1,B2,CCT |
| PvCO5 | 3021 | 1224 | 4 | 407 | B1,B2(S),CCT |
| PvCO6 | 2924 | 1353 | 5 | 450 | B1,B2(S),CCT |
| PvCO7 | 1059 | 903 | 3 | 301 | B1,B2,CCT |
| PvCO8 | 1078 | 981 | 2 | 326 | B1,B2,CCT |
| PvCO9 | 1859 | 1161 | 2 | 386 | B1,B2,CCT |
| PvCO10 | 10217 | 2085 | 8 | 694 | B1,B2,CCT |
| PvCO11 | 2309 | 1077 | 4 | 358 | B1,B2(S),CCT |
| PvCO12 | 2092 | 1185 | 4 | 394 | B1,B2(S),CCT |
| PvCO13 | 2316 | 1194 | 4 | 398 | B1,B2(S),CCT |
| PvCO14 | 1245 | 1071 | 3 | 356 | B1,B2,CCT |
| PvCO15 | 3234 | 996 | 2 | 326 | B1,B2,CCT |
| PvCO16 | 1300 | 1185 | 2 | 394 | B1,CCT |
| PvCO17 | 1343 | 1110 | 3 | 383 | B1,CCT |
| PvCO18 | 1316 | 1194 | 2 | 397 | B1,CCT |
| PvCO19 | 1072 | 891 | 3 | 296 | B1,B2,CCT |
