# Supplementary material for: Overexpression of PvCO1, a bamboo CONSTANS-LIKE gene, delays flowering by reducing expression of the FT gene in transgenic Arabidopsis
Source: BMC Plant Biol. 2018 Oct 12;18:232. doi: 10.1186/s12870-018-1469-0 (PMC6186071; doi:10.1186/s12870-018-1469-0)
Supplement: Supplementary file 4 — Table S4. Characterization of COL gene family members in Ph. violascens. (DOCX 23 kb) [file 12870_2018_1469_MOESM4_ESM.docx]

Table S4 Accession numbers of *COL* gene family members in *Arabidopsis*, *Oryza sativa* and *Ph. heterocycla*

| Gene name | Gene Symbol | Gene name | Gene Symbol | Gene name | Gene Symbol |
| --- | --- | --- | --- | --- | --- |
| *CO* | *NM_121589* | *OsA (Hd1)* | *Os06g16370* | *PhCO1* | *PH01002508* |
| *CO1* | *NM_121590* | *OsB (OsCO3)* | *Os09g06464* | *PhCO2* | *PH01001244* |
| *CO2* | *NM_111105* | *OsC* | *Os04g42020* | *PhCO3* | *PH01005551G0030* |
| *CO3* | *NM_128038* | *OsD* | *Os02g39710* | *PhCO4* | *PH01000780G0510* |
| *CO4* | *NM_122402* | *OsE* | *Os06g44450* | *PhCO5* | *PH01001120G0010* |
| *CO5* | *AY114006* | *OsF* | *Os02g08150* | *PhCO6* | *PH01001246G0150* |
| *CO6* | *AY081541* | *OsG* | *Os08g15050* | *PhCO7* | *PH01001462G0020* |
| *CO7* | *NM_106047* | *OsJ* | *Os03g50310* | *PhCO8* | *PH01000459G0310* |
| *CO8* | *NM_103803* | *OsK* | *Os02g49880* | *PhCO9* | *PH01000682* |
| *CO9* | *NM_111644* | *OsL* | *Os06g15330* | *PhCO10* | *PH01005551* |
| *CO10* | *AB023039* | *OsM* | *Os06g19444* | *PhCO11* | *PH01001639G0300* |
| *CO11* | *NM_117613* | *OsN* | *Os02g49230* | *PhCO12* | *PH01001710G0270* |
| *CO12* | *NM_113084* | *OsO* | *Os08g42440* | *PhCO13* | *PH01003421G0140* |
| *CO13* | *NM_130356* | *OsP* | *Os03g22770* | *PhCO14* | *PH01000481G0620* |
| *CO14* | *NM_128910* | *OsQ* | *Os09g33550* | *PhCO15* | *PH01000048G0270* |
| *CO15* | *NM_102570* | *OsR* | *Os07g47140* | *PhCO16* | *PH01000142G0230* |
| *CO16* | *NM_102355* | *OsS* | *Os06g01340* | *PhCO17* | *PH01001011G0420* |
|  |  |  |  | *PhCO18* | *PH01002146G0170* |
|  |  |  |  | *PhCO19* | *PH01002727G0080* |
