# Supplementary material for: Overexpression of PvCO1, a bamboo CONSTANS-LIKE gene, delays flowering by reducing expression of the FT gene in transgenic Arabidopsis
Source: BMC Plant Biol. 2018 Oct 12;18:232. doi: 10.1186/s12870-018-1469-0 (PMC6186071; doi:10.1186/s12870-018-1469-0)
Supplement: Supplementary file 5 — Figure S1. Alignment of predicted N and C amino acid sequences of partial COLs from Arabidopsis thaliana, Oryza sativa, Ph. violascens, and Ph. heterocycla. The B-box domain and CCT domain are labeled by red lines. The second diverged B-box in group II is labeled by red box. (DOCX 117 kb) [file 12870_2018_1469_MOESM5_ESM.docx]

B-box 2

B-box 1


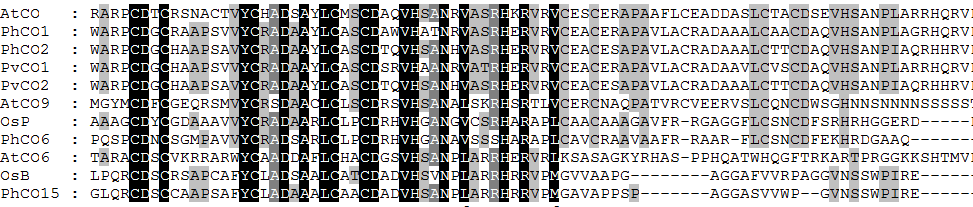


CCT domain

Group Ⅲ

GroupⅡ

GroupⅠ


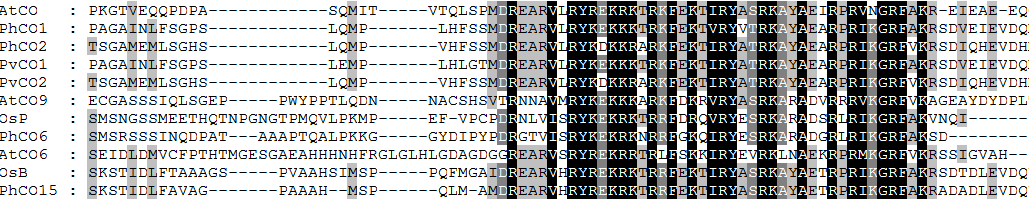


Group Ⅲ

GroupⅡ

GroupⅠ

Fig. S1. Alignment of predicted N and C amino acid sequences of partial COLs from *Arabidopsisthaliana, Oryza sativa, Ph. violascens,* and *Ph. heterocycla*. The B-box domain and CCT domain are labeled by red lines. The second diverged B-box in group II is labeled by red box.
